# Atualização no Tratamento da Hipertensão Arterial Pulmonar

**DOI:** 10.36660/abc.20200702

**Published:** 2021-10-06

**Authors:** Caio J. Fernandes, Daniela Calderaro, Ana Paula Luppino Assad, William Salibe-Filho, Luciana Tamie Kato-Morinaga, Susana Hoette, Bruna Piloto, Marcela Araújo Castro, Roberta Pontes Lisboa, Taysa Antonia Felix da Silva, Murillo de Araújo Martins, Jose L. Alves-Jr, Carlos Jardim, Mario Terra-Filho, Rogerio de Souza

**Affiliations:** 1 Incor Faculdade de Medicina Universidade de São Paulo São PauloSP Brasil Unidade de Circulação Pulmonar - Divisão de Pneumologia – Incor - Faculdade de Medicina da Universidade de São Paulo, São Paulo, SP - Brasil; 2 Instituto do Câncer Faculdade de Medicina Universidade de São Paulo São PauloSP Brasil Instituto do Câncer da Faculdade de Medicina da Universidade de São Paulo, São Paulo, SP - Brasil; 3 Hospital Sírio-Libanês São PauloSP Brasil Hospital Sírio-Libanês, São Paulo, SP - Brasil; 4 Incor Faculdade de Medicina Universidade de São Paulo São PauloSP Brasil Unidade de Medicina Interdisciplinar - Divisão de Cardiologia – Incor - Faculdade de Medicina da Universidade de São Paulo, São Paulo, SP - Brasil; 5 Hospital das Clínicas Faculdade de Medicina Universidade de São Paulo São PauloSP Brasil Disciplina de Reumatologia do Hospital das Clínicas da Faculdade de Medicina da Universidade de São Paulo, São Paulo, SP - Brasil

**Keywords:** Hipertensão arterial pulmonar, Hipertensão Pulmonar, Diagnóstico, Terapêutica

## Abstract

Muitos avanços ocorreram nas últimas décadas na terapêutica da hipertensão arterial pulmonar (HAP), uma doença grave, progressiva, incurável e potencialmente fatal. Para seu tratamento adequado, são fundamentais o diagnóstico hemodinâmico e a classificação de sua etiologia, em que várias delas (colagenoses, hipertensão portal, cardiopatia congênitas, esquistossomose) requerem medidas específicas, além do tratamento farmacológico característico para HAP. O tratamento com fármacos-alvo para HAP baseia-se em produtos farmacêuticos que interferem em três vias fisiopatológicas moleculares: da prostaciclina, da endotelina e do óxido nítrico. Tais fármacos apresentam múltiplas apresentações (oral, endovenosa, subcutânea e inalatória) e mudaram a história da HAP. Essas medicações e suas estratégias de uso, assim como particularidades das diferentes formas de HAP, são o foco desta revisão.

## Introdução

A hipertensão arterial pulmonar (HAP) é uma condição clínica que cursa com remodelamento do território vascular pulmonar, levando à obliteração dele e consequente aumento da resistência vascular.^1^ A elevação da resistência acarreta aumento das pressões do sistema, representando o aumento da carga imposta ao ventrículo direito, que evolui com progressiva insuficiência, sendo a principal causa dos sintomas associados à doença.^2^

Trata-se de uma situação clínica rara e grave, acometendo entre 2 a 5 pacientes por milhão de adultos por ano,^3^ com mediana de sobrevida de 2,8 anos na ausência de tratamento específico.^4^ Todavia, uma série de medicamentos, com múltiplas apresentações (oral, endovenosa, subcutânea e inalatória) foram desenvolvidos a partir da década de 1990, mudando sobremaneira a realidade da HP^5^ e seu impacto na qualidade de vida dos pacientes.^6^ Dados do registro francês de HAP demonstraram que após a introdução da terapêutica específica, a sobrevida dos pacientes passou a ser de 82,9% em 1 ano e de 58,2% em 3 anos, o que corresponde a uma melhoria estimada em pelo menos 15% quando comparada com a sobrevida prevista sem o acesso ao tratamento farmacológico.^7^ Tais medicamentos, suas estratégias de uso e as particularidades das diferentes formas de HAP são o foco desta revisão.

### Definições

**Figura 1:**
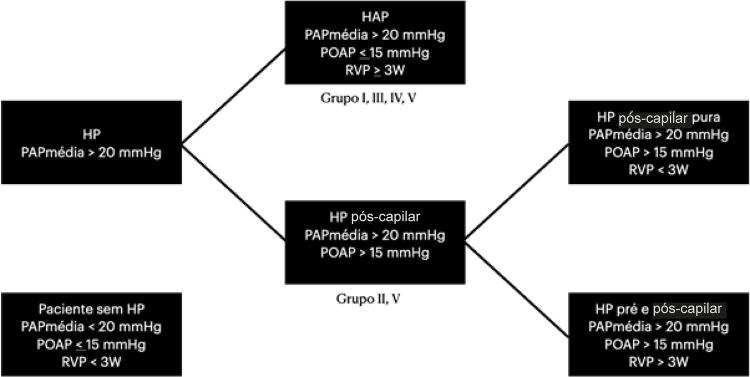
– *Definições de alterações hemodinâmicas do sistema vascular pulmonar e sua correlação com os grupos da classificação de hipertensão pulmonar. HP: hipertensão pulmonar; HAP: hipertensão arterial pulmonar; PMAP: pressão média de artéria pulmonar média; POAP: pressão de oclusão de artéria pulmonar; RVP: resistência vascular pulmonar; W: Woods; mmHg: milímetros de mercúrio.*

Define-se hipertensão pulmonar (HP) como uma condição em que a pressão média de artéria pulmonar (PMAP) é superior a 20 mmHg.^8^ Classicamente, utilizou-se o nível de 25 mmHg para defini-la,^9^ mas estudos recentes sugerem que níveis menores já estão associados ao pior prognóstico.^10^ Concomitantemente, se a pressão de oclusão de artéria pulmonar (POAP) for menor ou igual a 15 mmHg, temos a condição de HP pré-capilar; nessa situação, o predomínio da doença vascular está no território arterial. Quando a POAP for superior a 15 mmHg, temos a condição de HP pós-capilar, o que sugere a presença de alterações nas câmaras cardíacas esquerdas. Baseado nestas definições, representadas na Figura 1 , vê-se que o cateterismo cardíaco direito é fundamental para a caracterização adequada da HP.^11^ Vale a pena ressaltar que, enquanto os atuais critérios de HAP incluem valores de resistência vascular pulmonar (RVP) superiores a 3W, dados recentes indicam que valores superiores a 2,2W apresentam impacto negativo na sobrevida desta população e podem apresentar resposta ao tratamento clínico.^12^ É possível que futuras definições de HAP incluam, além do critério pressórico de 20 mmHg, valores de RVP superiores a 2,2W.

### Classificação

A partir das definições hemodinâmicas e agregando características fisiopatológicas, clínicas e de tratamento das diferentes etiologias causadoras de HP, é possível classificar os casos em 5 grupos distintos.^8 , 13^ Considera-se do grupo I os pacientes que tenham doença arterial pulmonar predominante, na ausência de doença pulmonar ou trombembólica, o foco desta revisão. O grupo II inclui pacientes cuja gênese da HP seja doença cardíaca esquerda e aumento da pressão hidrostática do sistema, a partir do átrio esquerdo.^14^ O grupo III inclui pacientes com HP por doença pulmonar crônica, nos quais a gênese da enfermidade advém da perda do leito vascular pulmonar e da vasoconstrição hipóxica.^15^ O grupo IV inclui pacientes com HP por embolia pulmonar crônica (HPTEC), e possui um manejo clínico distinto, além do escopo deste texto. Recomendações para diagnóstico e tratamento dos pacientes com HPTEC podem ser encontradas em outras publicações.^16^ O grupo V inclui pacientes com doenças mais raras, com múltiplos mecanismos.^8^ As diferentes etiologias de HP e sua classificação em grupos estão descritos na Tabela 1 . Vale ressaltar que, embora esteja fora do escopo desta revisão, o processo diagnóstico para correta classificação dos casos de HP é bastante extenso e abrangente, garantindo que as estratégias de tratamento sejam adequadas aos mecanismos fisiopatológicos predominantes como causa da elevação pressórica no território vascular pulmonar.^17^

**Tabela 1:** – Classificação das etiologias de HP em grupos (modificado de 5)

1. Hipertensão arterial pulmonar (HAP)
1.1 HAP idiopática
1.2 HAP hereditária
1.3 Induzida por fármacos ou toxinas
1.4 Associada a:
1.4.1 Doenças do tecido conectivo
1.4.2 Infecção por HIV
1.4.3 Hipertensão portal
1.4.4 Doenças cardíacas congênitas
1.4.5 Esquistossomose
1.5 Respondedores aos bloqueadores de canal de cálcio
1.6 Doença pulmonar veno-oclusiva e/ou hemangiomatose capilar pulmonar
1.7 Hipertensão pulmonar persistente do recém-nascido
**2. Hipertensão pulmonar por doença cardíaca esquerda**
2.1 Insuficiência cardíaca com FE preservada
2.2 Insuficiência cardíaca com FE reduzida
2.3 Doença valvar
2.4 Cardiopatias congênitas ou adquiridas que levam à HP pós-capilar
**3. Hipertensão pulmonar por doença pulmonar e/ou hipoxia**
3.1 Doença pulmonar obstrutiva
3.2 Doença pulmonar restritiva
3.3 Outras doenças pulmonares com distúrbio misto
3.4 Hipoxia sem doença estrutural pulmonar
3.5 Doenças do desenvolvimento pulmonar
**4. Hipertensão pulmonar por obstruções de artéria pulmonar**
4.1 Hipertensão pulmonar por trombembolismo pulmonar crônico
4.2 Outras obstruções de artéria pulmonar
**5. Hipertensão pulmonar por mecanismos multifatoriais e/ou desconhecidos**
5.1 Doenças hematológicas: anemia hemolítica crônica, doenças mieloproliferativas
5.2 Doenças sistêmicas e metabólicas: histiocitose pulmonar de células de Langerhans, doença de Gaucher, doenças de depósito do glicogênio, neurofibromatose e sarcoidose
5.3 Outras: mediastinite fibrosante, insuficiência renal crônica com ou sem hemodiálise
5.4 Cardiopatias congênitas complexas

*HP: hipertensão pulmonar; HAP: hipertensão arterial pulmonar; HIV: vírus da imunodeficiência humana; FE: fração de ejeção.*

### Tratamento da HAP

#### Medidas gerais

Após a confirmação diagnóstica da HAP, medidas gerais que objetivem minimizar as consequências da doença devem ser implementadas. Nesse contexto geral, três delas se destacam: evitar a gestação (condição associada a um agravamento da condição hemodinâmica, pela necessidade de aumento do débito cardíaco durante a gestação, resultando em elevada mortalidade materno-fetal),^18^ realizar imunização para influenza e pneumococo^9^ e o oferecer apoio psicossocial aos portadores de HAP.^19^

Além das que foram citadas, outras medidas que podem ser adotadas são: uso de diuréticos, administração suplementar de oxigênio e a não realização de exercícios físicos extenuantes. Exercícios físicos, com o objetivo de reabilitação, podem ser recomendados, mas sob supervisão,^20^ após o início de tratamento farmacológico específico.

Estudos da década de 1980^21^ sugeriam o benefício de anticoagulação, baseando-se nos fenômenos de trombose *in situ* presentes em estudos de biópsias pulmonares na HAP. No entanto, dados mais recentes^22^ sugerem benefício da anticoagulação apenas para pacientes com hipertensão arterial pulmonar idiopática (HAPI), hereditária ou associada ao uso de anorexígenos. Dessa forma, a indicação de anticoagulação em HAP deixou de ser uma conduta universal e passou a estar baseada, caso a caso, na avaliação de risco/benefício.^23^

#### Avaliação da vasorreatividade

Para pacientes com HAPI, hereditária ou induzida por fármacos, o teste de vasorreatividade deve ser realizado durante o cateterismo cardíaco direito diagnóstico. O padrão-ouro utiliza a inalação de óxido nítrico 10 a 20 ppm durante 10 minutos. O teste é positivo quando há uma queda maior de 10mmHg na PMAP, alcançando valores abaixo de 40mmHg e sem redução do débito cardíaco (DC).^2^ O teste de vasorreatividade visa a identificar um subgrupo de pacientes nos quais o aumento do tônus vascular seja o mecanismo predominante na gênese da HAP, não o remodelamento vascular.^24^

Pacientes com HAP que apresentem resposta positiva ao teste de vasorreatividade devem ser submetidos ao tratamento com bloqueador de canal de cálcio (BCC), preferencialmente de ação prolongada e com a maior dose tolerada.^25^ Sugere-se iniciar com 10mg de anlodipino 1x/dia, diltiazem 60mg 3x/dia ou nifedipina 30mg 2x/dia. Pacientes que não evoluam para classes funcionais (CF) I/ II e não apresentem melhora hemodinâmica devem receber medicação específica para HAP. Cerca de 12,6% dos pacientes diagnosticados com HAPI apresentam resposta aguda à vasodilatação; porém, metade não persiste com boa resposta clínica ao uso de BCC após um ano de seguimento.^26^ Pacientes que não tenham feito teste de vasorreatividade não devem ser tratados com BCC.^23^

## Tratamento específico

### Vias de tratamento

O principal achado anatomopatológico na HAP é o remodelamento vascular pulmonar, com espessamento das camadas íntima e camada média.^27^ A vasoconstrição também possui importante papel no desenvolvimento da doença, com aumento do tônus vascular e proliferação das células musculares lisas (CML) das arteríolas.^25^ Três vias fisiopatológicas relacionadas aos mecanismos moleculares que geram esses achados histológicos foram identificadas e passaram a ser alvos para o tratamento farmacológico da HAP: da prostaciclina, da endotelina e do óxido nítrico.^28^

### Via da prostaciclina

A prostaciclina (PGI_2_) é uma molécula derivada do ácido araquidônico, capaz de atuar em receptores transmembranas de múltiplos tecidos, com variadas ações biológicas.^29^ Na circulação pulmonar, ao estimular o receptor IP, a PGI_2_ é capaz de induzir o relaxamento das CML das arteríolas pulmonares, com consequente vasodilatação, e inibir a sua proliferação, ao estimular a produção do AMP cíclico.^30^ Por outro lado, outra molécula da via da PGI_2_, o tromboxano A_2_ (TXA_2_) contrabalança seu efeito, promovendo vasoconstrição e aumento da agregação plaquetária. Na HAP, os níveis de PGI_2_ e a atividade enzimática da prostaciclina sintase estão significativamente reduzidos, e seu equilíbrio pende em direção ao TXA_2_.^31^ Dessa forma, uma estratégia para o tratamento de HAP é atuar diretamente no receptor IP, com reposição direta da PGI_2_ (epoprostenol)^32^ ou com fármacos de estrutura análoga à da PGI_2_ (por exemplo: treprostinil, beraprost e iloprost).^33 , 34^ Existem, ainda, fármacos capazes de atuar no receptor IP, com estrutura molecular distinta da PGI_2_ (por exemplo, selexipague).^35^

### A via da endotelina

A endotelina-1 (ET1) é o mais potente vasoconstritor natural de sistemas biológicos,^36^ seus níveis são elevados tanto no endotélio vascular pulmonar quanto no sangue de pacientes com HAP.^37^ A ET1 age por meio de dois tipos de receptores: os receptores de endotelina A (ET_A_) e B (ET_B_). A bosentana^38^ e a macitentana^39^ são antagonistas dos receptores de endotelina (ARE), que bloqueiam de forma não seletiva os receptores A e B, enquanto a ambrisentana^40^ apresenta maior afinidade pelo receptor ET_A_. Todas as três substâncias são fármacos orais e mostraram-se eficientes no tratamento da HAP.

### A via do óxido nítrico

O NO é um potente vasodilatador endógeno, que atua nas CML vasculares através do estímulo da guanilil ciclase (GC), levando à produção do GMP cíclico.^41^ Pacientes com HAP apresentam níveis reduzidos de NO sérico e tecidual.^42^ A fosfodiesterase do tipo 5 (PDE-5) é a enzima responsável pela degradação do GMP cíclico. A inibição da PDE-5 leva a um aumento na concentração de GMP cíclico e ao consequente relaxamento das CML, promovendo vasodilatação.^43^ Sildenafil, tadalafil e vardenafil são os fármacos inibidores da PDE-5 (PDE-5i) disponíveis.

Atuando em outro ponto da via do NO, há o riociguate, um estimulante da GC. Ele potencializa a atividade da GC e a ativa de forma independente ao NO.^44^ A GC estimulada pelo riociguate potencializa a conversão de GTP em GMP cíclico, promovendo vasodilatação. O fármaco pode ser utilizado tanto em HAP^45^ quanto em HPTEC.^46^

Os fármacos disponíveis para o tratamento de HAP no Brasil estão descritos na Tabela 2 .

**Tabela 2:** – Fármacos autorizados e disponíveis no Brasil para o tratamento de HAP

Fármacos	Posologia	Via de administração	Efeitos colaterais mais frequentes	Via	Referência
Iloprost	2,5-5 mcg 6-9x ao dia	Inalatória	Tosse Efeitos irritativos locais	PGI_2_	^34^
Selexipague	200-1600 mcg 2x ao dia	Oral	Cefaleia Diarreia	PGI_2_	^35^
Ambrisentana	5-10 mg 1 x ao dia	Oral	Anemia Edema	ET1	^40^
Bosentana	62,5-125 mg 2x ao dia	Oral	Anemia Hepatotoxicidade	ET1	^38^
Macitentana	10 mg 1x ao dia	Oral	Anemia Hepatotoxicidade Edema	ET1	^39^
Sildenafil	20-80 mg 3x ao dia	Oral	Cefaleia	NO	^43^
Riociguate	0,5-2,5 mg 3x ao dia	Oral	Cefaleia Hipotensão	NO	^45^

*PGI_2_: prostaciclina; ET1: endotelina; NO: óxido nítrico.*

## Estratégias de tratamento

Ao longo dos últimos anos, houve significativa mudança na estratégia de uso dos diferentes fármacos disponíveis, com indicação cada vez mais precoce de tratamento e combinação de fármacos de diferentes vias/classes.

O uso combinado de fármacos apoia-se no potencial de sinergia existente na intervenção simultânea em diferentes vias fisiopatológicas. Comparada com a monoterapia, a associação adicional de um segundo medicamento (terapia combinada sequencial) mostrou-se benéfica para diferentes combinações de fármacos administrados.^35 , 39 , 47^ Tais resultados foram reforçados em uma metanálise de 14 estudos de terapia combinada sequencial e que mostrou redução de piora clínica quando comparada com a monoterapia.^48^

Em diferente abordagem, um grande estudo clínico avaliou o uso combinado de fármacos desde o diagnóstico, mais precisamente a associação de tadalafil e ambrisentana.^47^ Esta combinação levou a uma redução de 50% no desfecho combinado de piora clínica quando comparada com a terapia com qualquer um dos medicamentos isoladamente, sem diferença significativa em termos de efeitos colaterais.

**Figura 2:**
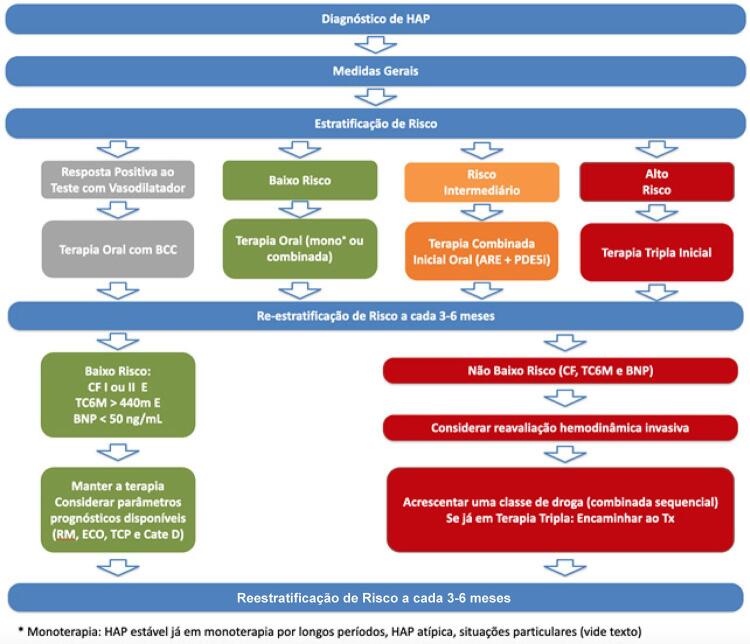
– *Algoritmo de tratamento e estratificação de risco no seguimento da HAP realizado pelo grupo de circulação pulmonar do Incor (USP).*

Embora não haja estudos comparando diretamente a combinação inicial *versus* sequencial, a evidência sugere que o tratamento combinado inicial seja bem tolerado e benéfico, mesmo em pacientes considerados de baixo risco.^47^ Dessa forma, a recomendação atual é de que a terapia combinada oral seja considerada ao diagnóstico, conforme detalhado na Figura 2 .

No entanto, para uma minoria de pacientes, a estratégia de monoterapia ainda pode ser indicada. Pacientes portadores de hipertensão portal, HIV, cardiopatia congênita complexa, doença veno-oclusiva ou aqueles com HAPI e alta probabilidade de insuficiência cardíaca esquerda, com fração de ejeção preservada, podem iniciar tratamento utilizando apenas uma classe de fármaco. Além disso, pacientes que são já usuários de monoterapia e estejam em estabilidade clínica por longos períodos não necessitam obrigatoriamente de combinação farmacológica.^23^

O potencial da combinação de três fármacos desde o diagnóstico também já foi avaliado. Um estudo retrospectivo francês avaliou a eficácia da combinação tripla (epoprostenol, bosentana e sildenafil) no tratamento inicial de pacientes recém-diagnosticados com formas graves de HAP, o que demonstrou melhora na classe funcional, na capacidade de exercício e nos parâmetros hemodinâmicos. O estudo mostrou ainda que a sobrevida dos pacientes com terapia tripla foi melhor do que a prevista pelo registro histórico francês, sugerindo que exista benefício a longo prazo com o uso desta abordagem.^49^

No entanto, para pacientes menos graves, não há evidência que comprove a eficácia da terapia tripla inicial. Um estudo recente avaliou o uso de selexipague, macitentana e tadalafil *versus* terapia dupla inicial com macitentana e tadalafil em 247 pacientes recém-diagnosticados com HAP.^50^ Não foi detectada diferença na melhora da RVP, na distância percorrida no teste de caminhada de 6 minutos (TC6M) e nos níveis de NT-proBNP. Além disso, efeitos adversos foram mais presentes nos pacientes em uso de terapia tripla.

Por outro lado, a combinação sequencial de três fármacos para o tratamento de HAP parece ser eficiente. A adição do selexipague ao tratamento já estabelecido de ARE e PDE5i foi associada à diminuição do número de hospitalizações e eventos de progressão de doença.^51^ O benefício identificado foi mais pronunciado em pacientes em classe funcional 2.

Outra potencial estratégia seria a substituição de medicações dentro de uma mesma via, caso o paciente não apresente resposta clínica satisfatória. Apesar de promissora, ela ainda carece de comprovação científica robusta e não deve ser realizada de forma rotineira.^52^

## Estratificação de risco e seguimento clínico

Avaliar o risco de progressão da HAP é fundamental para orientar seu tratamento. Estratégias de estratificação de risco de morte, avaliando a combinação de múltiplos marcadores ( Tabela 3 ) tanto ao diagnóstico quanto ao longo do tratamento, mostraram-se eficientes para predizer o curso clínico da doença.^53 - 55^

**Tabela 3:** – Fatores prognósticos na hipertensão arterial pulmonar (adaptada da referência 8)

Determinantes do prognóstico	Mortalidade estimada em 1 ano		

	Baixo risco: < 5%	Risco intermediário: 5-10%	Alto risco: > 10%
Sinais clínicos de insuficiência ventricular direita	Ausente	Ausente	Presente
Progressão dos sintomas	Não	Lenta	Rápida
Síncope	Não	Ocasionalmente*	Repetidamente**
CF OMS	I, II	III	IV
DC6M	> 440 m	165-440 m	< 165 m
Teste de exercício cardiopulmonar	Pico VO_2_ > 15 mL/min/kg (> 65% pred.) Alça VE/VCO_2_ <36	Pico VO_2_ 11-15 mL/min/kg (35-65% pred.) Alça VE/VCO_2_ 36-44,9	Pico VO_2_ < 11mL/min/kg (< 35% pred.) VE/VCO_2_ ≥ 45
Níveis plasmáticos de NT-proBNP	BNP < 50 ng/L NT-proBNP<300 ng/mL	BNP 50-300 ng/L NT-proBNP 300-1.400 ng/L	BNP > 300 ng/L NT-proBNP > 1.400 ng/L
Exames de imagem (ECO, ressonância magnética do tórax)	Área do AD < 18 cm^2^ Ausência de derrame pericárdico	Área do AD 18-26 cm^2^ Ausência ou mínimo derrame pericárdico	Área do AD > 26 cm^2^ Derrame pericárdico presente
Parâmetros hemodinâmicos	Pressão do AD < 8 mmHg IC ≥ 2,5 L/min/m^2^ SvO_2_ > 65%	Pressão do AD 8-14 mmHg IC 2,0-2,4 L/min/m^2^ SvO_2_ 60-65%	Pressão do AD > 14 mmHg IC < 2,0 L/min/m^2^ SvO_2_< 60%

* Síncope ocasional durante exercício brusco ou intenso, ou síncope ortostática ocasional em paciente previamente estável. ** Episódios repetidos de síncope, mesmo em atividade física leve ou regular. VO_2_ pico: consumo de oxigênio no pico do esforço; VE: ventilação; VCO_2_: volume expirado de CO_2_; BNP/NT-próBNP: peptídeo natriurético tipo B/fragmento N-terminal do peptídeo natriurético tipo B; AD: átrio direito; IC: índice cardíaco; SvO_2_: saturação venosa central de oxigênio.

Existem várias formas de avaliar o risco de progressão da HAP. Uma delas é através da atribuição de 1 a 3 pontos para cada variável, de acordo com a faixa de risco em que ela se encontra (baixo, intermediário ou alto, respectivamente). Para obtenção do risco global, divide-se a soma de todos os pontos pelo número de variáveis utilizadas. O número inteiro mais próximo determina o risco em baixo (1), intermediário (2) ou alto (3).^55^ Outra abordagem, mais simples, procura apenas identificar os pacientes que possam ser classificados como baixo risco, através da combinação dos seguintes parâmetros combinados: níveis de BNP inferiores a 50pg/mL, TC6M superior a 440m e CF menor ou igual a 2.^53^ Existe ainda uma terceira abordagem, derivada da calculadora de risco oriunda do registro americano REVEAL ( *Registry to Evaluate Early and Long-Term PAH Disease Management* ), baseada em até 12 variáveis e que foi recentemente atualizada.^56^

Embora seja claro que a avaliação mais abrangente possa ter superioridade em relação à avaliação simplificada, a disponibilidade dos diferentes exames necessários limita sua utilização. Dessa forma, a estratificação de risco aqui sugerida baseia-se no que deve ser considerado o mínimo necessário para o adequado manejo clínico dos pacientes com HAP, através da combinação entre CF, TC6M e BNP, para a identificação de baixo risco ou não baixo risco ( Figura 2 ). Conforme disponibilidade, exames de ressonância magnética, ecocardiograma e teste cardiopulmonar de esforço devem ser considerados, haja vista seu potencial papel adjuvante.^23 , 57^ O objetivo do tratamento da HAP é fazer com que o paciente permaneça ou atinja baixo risco de progressão de doença. Para isso, as diferentes estratégias já discutidas são utilizadas de acordo com a estratificação de risco encontrada.

## Particularidades das diversas formas de HAP

### HAP associada às doenças do tecido conjuntivo

A HAP é uma complicação conhecida em pacientes com doença do tecido conjuntivo (DTC). As principais DTCs associadas à HAP são esclerose sistêmica (ES), lúpus eritematoso sistêmico (LES) e doença mista do tecido conjuntivo (DMTC); em menor grau, dermatomiosite e síndrome de Sjögren.^58^ No Brasil, a HAP associada à DTC representa cerca de 25%^59^ dos casos de HAP. Nos países ocidentais, a ES é a principal doença associada à ocorrência de HAP, enquanto em países asiáticos é o LES.^60 - 62^

A prevalência de HAP em pacientes com ES é em torno de 10%,^63^ e no LES é de 4%.^64^ A presença de anticorpos antifosfolípideos, anti-RNP e anti-Ro são preditivos de HAP em pacientes com LES.^65^ Em contrapartida, em pacientes com ES, doença de longa evolução, presença de telangiectasias, positividade do anticentrômero e redução de DLCO são os principais fatores relacionados à HAP.^66^

A ES é uma doença representada pela associação de vasculopatia e fibrose tecidual.^67^ Clinicamente, apresenta uma alta frequência de doença pulmonar intersticial (até 50% dos pacientes)^68^ e acometimento cardíaco (50 a 80% dos pacientes), ocasionando, frequentemente, disfunção diastólica.^69^ Portanto, nestes pacientes, a HP pode ser resultado de uma doença vascular pulmonar isolada ou associada aos diferentes mecanismos fisiopatológicos relacionados à HP dos grupos 2 e 3.^68^ Além disso, esses pacientes possuem risco aumentado de trombembolismo pulmonar e também podem apresentar doença veno-oclusiva associada, impactando ainda mais o seu prognóstico.^68^ É imperativo tentar determinar qual é o mecanismo preponderante, pois é o fator que ditará o tratamento.

Em pacientes com ES, é recomendada triagem anual de HP, mesmo nos assintomáticos.^70^ Os modelos de triagem compostos com fatores de risco, DLCO e BNP parecem ser mais sensíveis do que o uso isolado do ecocardiograma.^71^ No LES, diante da baixa prevalência de HAP, o rastreamento não é recomendado como rotina, devendo o ecocardiograma ser realizado apenas na presença de sintomas ( Tabela 4 ).

**Tabela 4:** Fatores de risco e rastreamento de HAP em pacientes com doença do tecido conjuntivo

	Fator de risco	Rastreamento
ES	Doença de longa evolução Telangiectasias Anticentrômero Redução de DLCO ou relação CVF/DLCO>1.6	Anual em assintomáticos ECO ± biomarcadores/ PFP ou algoritmo DETECT BNP/NT-próBNP e PFP ± ECO
LES	Serosite Anticorpos: antifosfolípideos, RNP e Ro	Apenas em sintomáticos

*ES: esclerose sistêmica; LES: lúpus eritematoso sistêmico; PFP: Prova de função pulmonar; DLCO: capacidade de difusão pulmonar para o monóxido de carbono; capacidade vital forçada (CVF); BNP/NT-próBNP: peptídeo natriurético tipo B/fragmento N-terminal do peptídeo natriurético tipo B.*

Diante da suspeita clínica, o fluxograma diagnóstico é o mesmo, objetivando confirmar HAP (grupo 1) e descartar outras formas de HP. O teste de vasorreatividade não é aplicado aos pacientes com DTC, uma vez que menos de 1% apresentam resposta sustentada.

Nos pacientes com formas mais inflamatórias como LES ou DMTC-HAP, pode optar-se pela imunossupressão antes do início de terapia específica. Em pacientes em classes funcionais I e II, recomenda-se o início de tratamento apenas com ciclofosfamida (CFF) e glicocorticoide (GCT). Nos casos em classes funcionais III e IV, a chance de resposta apenas com imunossupressores é menor, e recomenda-se uso de CFF e GCT associados à terapia específica, com reavaliação após 6 meses de CFF ( Tabela 5 ).^58 , 72^ Nos pacientes com ES, não há evidência de melhora com uso de imunossupressor.^73^

**Tabela 5:** Tratamento da HAP associada às doenças do tecido conjuntivo

	Imunossupressão (IS)	Terapia específica
ES	Não recomendada	Conforme estratificação de risco
LES classe funcional I/II	CFF e GCT por 6 meses	Quando mantido HP após IS
LES classe funcional III/IV	CFF e GCT associada à terapia específica	Associada a IS, conforme estratificação de risco
DMTC classe funcional I/II	CFF e GCT por 6 meses	Quando mantido HP após IS
DMTC classe funcional III/IV	CFF e GCT associada à terapia específica	Associada a IS, conforme estratificação de risco

*ES: esclerose sistêmica; LES: lúpus eritematoso sistêmico; DMTC: doença mista do tecido conjuntivo CFF: ciclofosfamida; GCT: glicocorticoide ; HP: hipertensão pulmonar.*

### HAP associada ao HIV

A associação entre HIV e HAP foi descrita pela primeira vez em 1987.^74^ Atualmente, estima-se uma prevalência de 0,5% de HAP nos pacientes portadores de HIV,^75^ sendo uma etiologia bastante relevante nos centros de referência. Em um registro brasileiro, 4,5% dos pacientes com HAP eram portadores de HIV.^59^

Os mecanismos fisiopatológicos envolvidos no quadro de HIV e HAP ainda não estão totalmente esclarecidos. Do ponto de vista histológico, em até 78% dos pacientes foi observado fibrose concêntrica da íntima, hipertrofia da média e lesões plexiformes, algo muito semelhante as causas não infeciosas de HAP.^76^

A presença de HAP confere pior prognóstico ao paciente com HIV, tendo a contagem de CD4^+^ menor que 200 células/µL e débito cardíaco menor que 2,8 L/min por m^2^ como preditores independentes de sobrevida.^77^ Recomenda-se, portanto, o tratamento com a terapia antirretroviral (TARV) para todos os pacientes com HIV, independente da contagem de CD4^+^ e carga viral,^78^ e da HAP concomitante.

Considerando a via do óxido nítrico, não existem estudos controlados com sildenafil que avaliem a resposta ao tratamento nos pacientes com HIV-HAP, e os resultados positivos encontrados com capacidade de exercício, CF e hemodinâmica derivam de estudos de casos.^79^ Sugere-se cautela, devido à interação com a TARV, especialmente com os inibidores de protease (IPr).^80^ Também não há estudos específicos com o riociguate, apesar de publicação recente que demonstrou segurança e tolerabilidade da associação entre este fármaco e a TARV.^81^

Entre os AREs, a bosentana foi avaliada para o tratamento de portadores de HVI-HAP em um estudo prospectivo com 59 pacientes.^82^ Foi demonstrado melhora no TC6M, nos sintomas e em dados hemodinâmicos. Nos estudos pivotais que avaliaram o uso de ambrisentana^40^ e macitentana,^39^ foram incluídos 17 e 10 pacientes com HIV-HAP, respectivamente, e não se encontraram interações com a TARV.^78^

Pequenas séries de casos avaliaram alguns prostanoides para o tratamento de HIV-HAP. O epoprostenol melhorou os dados hemodinâmicos em 6 pacientes.^83^ O iloprost mostrou efeito na melhora da CF e do TC6M de 4 pacientes.^84^ O selexipague foi administrado em 10 pacientes portadores de HIV no seu estudo pivotal.^35^ Não houve interação de selexipague com a TARV, nem necessidade de mudança de dose em um estudo de pacientes saudáveis em uso de selexipague 400 mcg uma vez ao dia e de dois antirretrovirais (lopinavir/ritonavir).^85^

### Hipertensão portopulmonar

A hipertensão portopulmonar (HPOP) é uma forma de HAP que surge na vigência de hipertensão portal que gera a abertura de *shunts* portossistêmicos e um aumento do fluxo sanguíneo na circulação pulmonar. Esse hiperfluxo acarreta disfunção e remodelamento endotelial, com aumento da produção de ET1 e elevação da pressão no território vascular pulmonar.^86^

Estima-se que aproximadamente 10,6% dos pacientes com HAP possuem HPOP.^87^ Por outro lado, HPOP está presente em 8,5% dos pacientes avaliados para transplante hepático.^88^ A HPOP não possui relação com a etiologia de hipertensão portal ou com a gravidade da doença hepática apresentada.^89^

A apresentação clínica dos pacientes é semelhante a outras formas de HAP, e o sintoma mais frequente é a dispneia. A ascite em paciente com HAP pode ser indicativa da presença de HPOP.^90^ O diagnóstico dessa condição envolve a realização de cateterismo direito para a confirmação de HAP, conforme previamente descrito, e a confirmação de hipertensão portal, via medida do gradiente de pressão trans-hepática superior a 4 mmHg.^91^

O tratamento de HPOP foi avaliado através de pequenos estudos, que demonstraram benefício das três classes de fármacos alvo para HAP. A hepatotoxicidade dos AREs não parece ser um problema maior para essa população, desde que monitorizados adequadamente.^92^ O maior estudo realizado em pacientes com HPOP demonstrou melhora hemodinâmica após 12 semanas de uso de macitentana, sem efeitos colaterais hepáticos.^93^ Alguns fármacos bem estabelecidos para o tratamento de hipertensão portal, como os betabloqueadores, não devem ser administradas em HPOP, por gerarem piora hemodinâmica e na capacidade de exercício.^94^

Vale a pena ressaltar o papel da HPOP nos pacientes em avaliação para transplante hepático. A HPOP é um marcador de pior prognóstico perioperatório neste procedimento.^91^ Valores de PMAP maiores do que 50mmHg contraindicam o transplante, e menores que 35mmHg indicam baixo risco de morte na realização do procedimento.^91^ Dessa forma, é fundamental a realização de um ecocardiograma para rastreamento de HAP em pacientes na fila para transplante hepático, ou em pacientes com hipertensão portal e dispneia, mesmo que de pequena intensidade.^95^

### Esquistossomose

A esquistossomose é uma doença parasitária endêmica no Brasil, relacionada a pobreza e más condições sanitárias, sendo causada por um verme trematódeo do gênero *Schistosoma* , cuja transmissão já foi relatada em mais de 70 países. A manifestação mais comum da esquistossomose crônica é a sua forma hepatoesplênica.^96^ No entanto, uma das suas manifestações mais graves e limitantes advém do acometimento da circulação pulmonar, com a presença de HAP.

Cerca de 5% dos pacientes com a forma hepatoesplênica da esquistossomose apresentam HAP.^96 , 97^ Assim, dada a alta prevalência mundial da esquistossomose, a HAP-Sch é potencialmente uma das formas mais prevalentes de HAP no mundo, particularmente em países emergentes.^97^ Um registro recente demonstrou que a HAP-Sch pode representar cerca de 20% dos casos incidentes de HAP.^59^

O diagnóstico de HAP-Sch requer a confirmação invasiva de HAP (vide a sessão definições) e de esquistossomose hepatoesplênica, mediante a presença de alterações ultrassonográficas compatíveis com a doença (presença de fibrose periportal), mais um dos três fatores epidemiológicos que associem a esquistossomose à condição ultrassonográfica encontrada: 1) paciente proveniente de área endêmica para a doença; 2) tratamento prévio para esquistossomose; e 3) presença de ovos do parasita no exame de fezes ou na biópsia retal.^98^

A HAP-Sch possui melhor curso clínico que a HAPI, mesmo na ausência de terapia específica; entretanto, isso não faz com que a doença seja isenta de risco. A taxa de mortalidade associada a HAP-Sch pode chegar até 15% em 3 anos.^99^ Casos de HAP com pronunciada dilatação das artérias pulmonares devem suscitar a hipótese de HAP-Sch, principalmente em regiões altamente prevalentes para essa condição.^100^

O tratamento de HAP-Sch é bastante semelhante ao de outras formas de HAP. Dados coletados em séries de casos demonstraram benefícios clínicos, hemodinâmicos^101^ e de sobrevida com o tratamento específico;^97^ assim, recomenda-se também seguir o algoritmo de tratamento proposto na Figura 2 para esta forma de HAP. A anticoagulação deve ser evitada, pelo risco de hemorragia digestiva. Todos os pacientes devem receber pelo menos um ciclo do tratamento parasitário, já que não se sabe se a persistência da infecção pode contribuir com a progressão do quadro vascular pulmonar.^97^

### Cardiopatia congênita

A HAP é uma condição relativamente frequente nos pacientes portadores de cardiopatias congênitas (CG), acometendo de 5-10% desta população.^102^ Tais cardiopatias podem gerar hiperfluxo na circulação pulmonar, induzindo a remodelamento endotelial e consequente da RVP, histologicamente indistinguível de outras formas de HAP. Dessa forma, a maioria das situações de HAP-CG são classificadas como grupo 1. Nesse grupo, quando a RVP supera a RVS e há a inversão do fluxo sanguíneo pelo *shunt* , caracteriza-se a síndrome de Eisenmenger.^103^ Esses pacientes possuem hipoxemia grave e, consequentemente, alterações hematológicas (eritrocitose e plaquetopenia). Pacientes com Eisenmenger também podem apresentar hemoptise, acidente vascular cerebral (AVC), abscessos cerebrais e maior incidência de morte súbita.^9^

O tratamento de HAP-CG segue as orientações previamente descritas para outras formas do grupo 1. Quando os sintomas de hiperviscosidade estiverem presentes na síndrome de Eisenmenger (geralmente com hematócrito > 65%), a flebotomia terapêutica pode ser realizada.^9^ Assim como em outras formas de HAP, a anticoagulação na síndrome de Eisenmenger é controversa,^104^ devendo ser ponderada caso a caso. O benefício do uso de oxigênio suplementar em pacientes com síndrome de Eisenmenger e hipoxemia é questionável,^105^ e sua indicação deve ser feita de forma individualizada.

Do ponto de vista de tratamento específico, o único fármaco validado de forma prospectiva na HAP-CG foi a bosentana, que melhorou o TC6M e diminui a RVP após 16 semanas de tratamento em pacientes com CF III.^106^ Séries menores demonstraram benefício com PDE5i e prostanoides.^9^

Algumas CGs como patência do ducto arterioso, defeito do septo interatrial do tipo seio venoso ou anormalidades de drenagem das veias pulmonares podem passar despercebidas em pacientes com HAP e serem erroneamente classificados como HAPI. No entanto, a identificação das CGs no contexto de HAP pode indicar a necessidade de correção cirúrgica do defeito, a depender da RVP ao diagnóstico^107^ e do comportamento clínico. Esse tipo de avaliação deve ser feito em centros com experiência nessa condição, a fim de indicar adequadamente a correção ou não. Apenas a possibilidade de correção já ressalta o quão fundamental é investigar ativamente a presença de CG. Uma estratégia de investigação das CGs em pacientes sob avaliação diagnóstica de HAP é a realização de ecocardiograma com microbolhas, buscando *shunts* intra e extracardíacos.

Algumas cardiopatias congênitas complexas, mais raras, são classificadas como grupo 5, de mecanismos incertos ou multifatorial.^8^ Nesses casos, dada a ausência e estudos prospectivos a abordagem farmacológica ou cirúrgica (incluindo transplantes de coração-pulmão) deve ser realizada de forma individualizada.

## Situações especiais

### Hipertensão pulmonar e gestação

As mudanças fisiológicas ao longo da gestação, como o aumento do volume sanguíneo e do DC,^108 , 109^ geralmente são mal toleradas pelas pacientes com HAP. Dessa maneira, a gravidez nessa condição é associada a elevadas taxas de morbimortalidade, oscilando entre 30 a 56%.^18^

Dada a alta letalidade da HAP em gestantes, recomenda-se a utilização de dois métodos contraceptivos. Não há consenso sobre o melhor método: os de barreira são seguros, porém a eficácia é diretamente relacionada ao uso adequado.^108^ Anticoncepcionais hormonais com progesterona isolada são efetivos, evitando a associação aos estrógenos, que podem aumentar os riscos de eventos trombembólicos.^109^ Dispositivos intrauterinos são uma opção, mas podem levar a reações vasovagais no momento da inserção.^110^

Caso a gestação ocorra, a mulher deve ser informada sobre os riscos potenciais. Em casos mais extremos, pode-se, inclusive, considerar a possibilidade de interrupção terapêutica da gestação.^109^ Caso esta seja a decisão, o aborto deve ser realizado preferencialmente até a 22ª semana.^111^ Em um registro europeu recente de HP e gestação,^112^ o aborto terapêutico foi realizado em 4% dos casos; em contrapartida, a mortalidade perinatal nas pacientes com HAPI foi de 43%. Não há consenso sobre a melhor via de parto, mas há a tendência em realizar a cesariana entre 32 a 36 semanas,^111^ evitando-se a anestesia geral.^113^ Pré-eclampsia ou eclampsia, parto prematuro, morte fetal e sangramento pós-parto foram as complicações mais frequentes nas gestantes com HP.^114^

O tratamento específico de HAP deve ser adaptado caso a paciente engravide e opte pela manutenção da gestação até o termo. Algumas séries de casos demonstraram eficácia na manutenção do uso de sildenafil e análogos da PGI2 na gestação.^18 , 113 , 115^ No entanto, os AREs devem ser evitados por conta de seu potencial efeito teratogênico.^116^

### Angina e HAP

**Figura 3:**
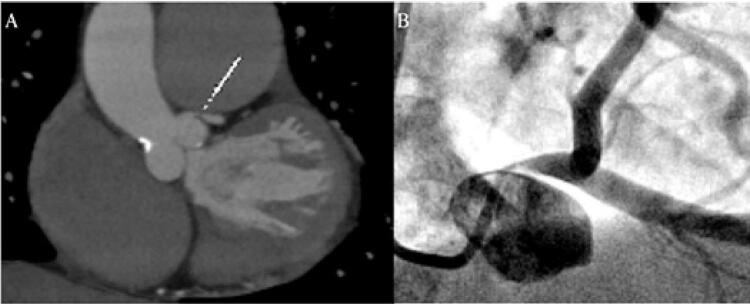
– *A) Angiotomografia coronária evidenciando deslocamento inferior do tronco da artéria coronária esquerda (TCE) por artéria pulmonar aneurismática e redução do calibre do óstio do TCE. B) Cineangiocoronariografia do mesmo paciente, evidenciando a suboculsão do óstio do TCE.*

A angina é referida por 15,8%^117^ a 29%^9^ dos pacientes com HAP, e pode decorrer do desequilíbrio entre a oferta e o consumo miocárdico de oxigênio no ventrículo direito, sem alteração de fluxo em coronárias epicárdicas. O aumento da tensão da parede ventricular acarreta redução da reserva de fluxo coronária, o que associado ao maior consumo de oxigênio pelo ventrículo, com aumento do seu trabalho e hipertrofia miocárdica, já justificaria a isquemia. Entretanto, outra consequência da HAP é o remodelamento vascular, com aumento do diâmetro de artérias pulmonares. Por sintopia, a artéria pulmonar dilatada e com regime hipertensivo pode determinar deslocamento inferior do tronco da artéria coronária esquerda (TCE) que se aproxima do seio coronário esquerdo ( Figura 3 ). Essa compressão pode acarretar importante diminuição de calibre do óstio do TCE.^118^ Em pacientes com angina e HAP, é descrita prevalência de até 40% de obstrução significativa em TCE por compressão extrínseca.^117^ O maior preditor de compressão de TCE nesses pacientes foi o diâmetro de artéria pulmonar > 40 mm, seguido da relação artéria pulmonar/aorta ≥1,5.^117^

O impacto prognóstico da compressão do TCE na HAP ainda é incerto. O manejo terapêutico dessa compressão é feito analogamente ao que se faz em lesão de TCE por aterosclerose: a revascularização miocárdica deve ser considerada, sendo a abordagem percutânea a modalidade mais atrativa e segura para esses pacientes.^119^ Dados italianos demonstraram um bom resultado com essa abordagem. Entre 53 pacientes submetidos a angioplastia e com tempo médio de seguimento de 4,5 anos, 19 faleceram (37,3%) sem nenhum caso de infarto ou trombose de *stent* , sendo que 5 pacientes necessitaram nova angioplastia.^120^

A compressão de TCE deve ser considerada em todos os pacientes com hipertensão pulmonar e angina, com investigação inicial pela angiotomografia coronária. Pacientes em programação de realizar cateterismo direito e com alta probabilidade clínica de obstrução extrínseca de TCE (por exemplo, angina em pacientes com grandes aneurismas de artéria pulmonar) podem fazer a cineangiocoronariografia diretamente, mesmo sem a angiotomografia de coronárias. Ressalta-se que, diante do aumento da sobrevida em pacientes com HAP, a comorbidade aterosclerótica também deve ser considerada, sendo importante o controle dos fatores tradicionais de risco cardiovascular.

### Arritmias supraventriculares e HAP

As arritmias cardíacas são frequentes em pacientes com HAP; notadamente as taquicardias supraventriculares, quer por exacerbação de automatismo, quer por mecanismos de reentrada. Estudos retrospectivos reportam incidência anual de taquicardia supraventricular sustentada entre 2,8%^121^ e 3,5%;^122^ entretanto, tais taxas nitidamente subestimam a real incidência, dada a falta de estudos prospectivos com busca sistemática, e estratégia de monitorização contínua ou de demanda acessível.

A sobrecarga pressórica em câmaras direitas, o remodelamento cardíaco e o elevado tônus adrenérgico associados a hipertensão pulmonar propiciam essas arritmias. Hipocalemia e hipomagnesemia, comumente desencadeadas pelo uso crônico de diuréticos, também podem ser gatilhos.

As arritmias sustentadas mais comuns são a fibrilação atrial e o *flutter* atrial; porém, taquicardia por reentrada nodal e taquicardia atrial sustentada também são observadas. A maioria dos pacientes apresenta piora de classe funcional com os episódios de arritmia, e a persistência da arritmia confere maior mortalidade.^121^ O tratamento tem como alvo preferencial a restauração do ritmo cardíaco por meio do uso de fármacos de pouca influência inotrópica (como a amiodarona), cardioversão elétrica programada e, em casos selecionados, estudo eletrofisiológico e ablação. Esta última técnica deve ser precocemente considerada para os pacientes com *flutter* atrial. No preparo para cardioversão, ou em casos de recorrência e falha da mesma, o controle da resposta ventricular é fundamental para assegurar o débito cardíaco, devendo-se individualizar a terapia de acordo com a reserva funcional do ventrículo direito. Pacientes com disfunção importante de ventrículo direito por hipertensão pulmonar toleram mal betabloqueadores, e mesmo o uso de bloqueadores de canal de cálcio podem causar descompensação clínica. Nessas situações limítrofes, a utilização de digoxina e da própria amiodarona se torna uma opção. Cabe ainda ressaltar que todo paciente com hipertensão pulmonar e fibrilação atrial, ou *flutter* , precisa receber terapia anticoagulante, pois o risco de trombembolismo sistêmico e cerebral é alto.

## Conclusão

Muitos avanços ocorreram nas últimas décadas na terapêutica da HAP, uma doença grave, progressiva, incurável e potencialmente fatal. Para seu tratamento adequado, é fundamental o diagnóstico hemodinâmico e a classificação da sua etiologia. Várias etiologias (colagenoses, hipertensão portal, cardiopatia congênitas, esquistossomose) requerem medidas específicas, além do tratamento farmacológico específico para HAP.

O tratamento específico para HAP baseia-se em fármacos que interferem em três vias fisiopatológicas: da prostaciclina, a da endotelina e a do óxido nítrico. Atualmente, recomenda-se que o tratamento inicial inclua uma combinação de duas terapias orais e que seja aumentado caso o paciente não alcance os alvos terapêuticos desejados, estes determinados através da estratificação de risco de morte cardiovascular. Complicações cardiovasculares de HAP (compressão de tronco de coronária esquerda, arritmias supraventriculares) são frequentes e devem ser prontamente identificadas e tratadas, já que impactam a qualidade de vida e, potencialmente, o prognóstico de pacientes com HAP.
