# Supplementary material for: Atualização no Tratamento da Hipertensão Arterial Pulmonar
Source: Arq Bras Cardiol. 2021 Oct 6;117(4):750–64. [Article in Portuguese] doi: 10.36660/abc.20200702 (PMC8528352; doi:10.36660/abc.20200702)
Supplement: Translation

# Translation: Update on the Treatment of Pulmonary Arterial Hypertension

**Authors:** Caio J. Fernandes, Daniela Calderaro, Ana Paula Luppino Assad, William Salibe-Filho, Luciana Tamie Kato-Morinaga, Susana Hoette, Bruna Piloto, Marcela Araújo Castro, Roberta Pontes Lisboa, Taysa Antonia Felix da Silva, Murillo de Araújo Martins, Jose L. Alves-Jr, Carlos Jardim, Mario Terra-Filho, Rogerio de Souza

**Affiliations:** 1 Brasil Unidade de Circulação Pulmonar - Divisão de Pneumologia – Incor - Faculdade de Medicina da Universidade de São Paulo, São Paulo, SP - Brasil; 2 Brasil Instituto do Câncer da Faculdade de Medicina da Universidade de São Paulo, São Paulo, SP - Brasil; 3 Brasil Hospital Sírio-Libanês, São Paulo, SP - Brasil; 4 Brasil Unidade de Medicina Interdisciplinar - Divisão de Cardiologia – Incor - Faculdade de Medicina da Universidade de São Paulo, São Paulo, SP - Brasil; 5 Brasil Disciplina de Reumatologia do Hospital das Clínicas da Faculdade de Medicina da Universidade de São Paulo, São Paulo, SP - Brasil

**Keywords:** Pulmonary Arterial Hypertension, Pulmonary Hypertension, Diagnosis, Therapeutics

## Abstract

In the last decades, important advances have been made in the treatment of pulmonary arterial hypertension (PAH), a severe, progressive, incurable, and potentially fatal disease. For an adequate therapy, correct hemodynamic diagnosis and etiology classification are fundamental. Many etiologies – rheumatic disease, portal hypertension, congenital heart diseases, schistosomiasis – require specific measures, in addition to drug therapy for PAH. The specific therapy for PAH is based on medications that act on three pathophysiological pathways – prostacyclin, endothelin, and nitric oxide pathways. These drugs have multiple presentations (oral, intravenous, subcutaneous, and inhaled) and have changed the history of PAH. This review presents an overview of drug therapy strategies and different forms and peculiarities of PAH.

## Introduction

Pulmonary arterial hypertension (PAH) is a clinical condition that leads to remodeling obliteration of the pulmonary vascular bed, ultimately resulting in increased vascular resistance.^1^ This causes an increase in systemic pressure and right ventricular overload, which progresses to a gradual ventircular failure, which is the main cause of the symptoms associated with the disease.^2^

PAH is a rare and severe condition that affects 2-5 million patients per million adults per year,^3^ with a median survival of 2.8 years in the absence of specific treatment.^4^ However, since the 90’s, a number of medications with multiple presentations (oral, intravenous, subcutaneous and inhaled) have been developed and drastically changed the PAH course and the quality of life of these patients.^6^

Data from the French Pulmonary Hypertension Network registry showed that, after introduction of specific therapy, patients’ survival increased to 82.9% in one year and 58.2% in three years, corresponding to an estimated improvement of at least 15% as compared with the predicted survival in case of no access to pharmacological treatment.^7^ The type of these medications and their strategy use, as well as the different forms of PAH are the focus of this review.

### Definitions

Pulmonary hypertension (PH) is defined as an increase of mean pulmonary arterial pressure (MPAP) greater than 20 mmHg.^8^ Although a threshold of 25 mmHg had been classically used in PH definition,^9^ evidence from recent studies has supported that lower levels are already associated with a worse prognosis.^10^ A concomitant pulmonary artery occlusion pressure (PAOP) equal to or lower than 15 mmHg characterizes the pre-capillary PH; in this situation, vascular disease is predominantly in the arterial territory. A PAOP higher than 15 mmHg determines the presence of post-capillary PH, suggesting changes in the left heart chamber ( Figure 1 ). Based on this definition, it seems clear that right cardiac catheterization is fundamental for an adequate characterization of PH.^11^

**Figure 1:** – *Definitions of hemodynamic changes of pulmonary vascular system and its correlation with pulmonary hypertension classification groups. PH: pulmonary hypertension; PAH: pulmonary arterial hypertension; MPAP: mean pulmonary arterial pressure; PAOP: pulmonary artery occlusion pressure; PVR: pulmonary vascular resistance; W: Woods, mmHg: millimeter of mercury.*

It is worth pointing out that, while current criteria for PAH definition include pulmonary vascular resistance (PVR) values greater than 3.0 W, recent data have indicated that PVR values greater than 2.2 W already have an impact on patients’ survival and a response to medical treatment.^12^ Thus, it is possible that future definitions of PAH will include PVR values greater than 2.2 W in addition to the criterion for MPAP greater than 20 mmHg.

### Classification

Based on hemodynamic definitions, and also considering pathophysiological, clinical and therapeutical features of different etiologies of PH, the cases of this disease can be classified into five groups.^8 , 13^ Group I, patients with predominant pulmonary arterial disease, in the absence of pulmonary or thromboembolic disease (focus of this revision); group II, patients with PH caused by left heart disease and elevation in hydrostatic pressure from the left atrium.^14^ Group III includes patients with PH associated with chronic pulmonary disease, caused by loss of pulmonary vascular bed and hypoxic vasoconstriction.^15^ Group IV includes patients with pulmonary hypertension due to chronic pulmonary embolism, and has a distinct clinical management, beyond the scope of this text. Recommendations for the diagnosis and treatment of patients with pulmonary hypertension due to chronic pulmonary embolism can be found in other publications.16 Group V includes patients with rarer diseases, involving multiple mechanisms.^8^ The different etiologies of PH and their classifications are described in Table 1 . Of note, although out of the scope of this review, the diagnostic process for a correct classification of PH cases is extensive and comprehensive, allowing the provision of appropriate treatment strategies to the predominant pathophysiological mechanisms of pulmonary vascular pressure elevation.^17^

**Table 1:** – Classification of pulmonary hypertension etiologies by groups (modified from reference 5)

1. Pulmonary arterial hypertension (PAH)
1.1 Idiopathic PAH
1.2 Heritable PAH
1.3 Drug- or toxin-induced PAH
1.4 PAH associated with:
1.4.1 Connective tissue disease
1.4.2 HIV infection
1.4.3 Portal hypertension
1.4.4 Congenital heart diseases
1.4.5 Schistosomiasis
1.5 Calcium channel blocker responders
1.6 Pulmonary veno-occlusive disease and/or pulmonary capillary hemangiomatosis
1.7 Persistent pulmonary hypertension of the newborn
**2. Pulmonary hypertension (PH) due to left heart disease**
2.1 Heart failure with preserved ejection fraction
2.2 Heart failure with reduced ejection fraction
2.3 Valvular heart disease
2.4 Congenital or acquired heart diseases leading to post-capillary pulmonary hypertension
**3. Pulmonary hypertension due to pulmonary disease and/or hypoxia**
3.1 Obstructive lung disease
3.2 Restrictive lung disease
3.3 Other lung diseases with mixed obstructive/restrictive pattern
3.4 Hypoxia without structural lung disease
3.5 Developmental lung diseases
**4. Pulmonary hypertension due to pulmonary artery obstruction**
4.1 Pulmonary hypertension due to chronic pulmonary thromboembolism
4.2 Other pulmonary artery obstructions
**5. Pulmonary hypertension with multifactorial and/or unknown mechanisms**
5.1 Hematological diseases: chronic hemolytic anemia, myeloproliferative disorders
5.2 Systemic and metabolic diseases: pulmonary Langerhans cell histiocytosis, Gaucher’s disease, glycogen storage disease, neurofibromatosis and sarcoidosis
5.3 Others: fibrosing mediastinitis, chronic renal failure, with or without hemodialysis
5.4 Complex congenital heart diseases

*PH: pulmonary hypertension; PAH: pulmonary arterial hypertension; HIV: human immunodeficiency virus.*

### Treatment of PAH

#### General measures

After the diagnosis of PAH is confirmed, general measures aiming at minimizing the consequences of the disease should be implemented. In this context, three important measures should be considered: to prevent pregnancy (which is associated with a worsening of hemodynamic status due to cardiac output increase, leading to elevated maternal-fetal mortality),^18^ immunization against influenza and pneumococcal disease,^9^ and psychosocial support to PAH patients.^19^ Other measures include the use of diuretics, supplemental oxygen, and avoidance of strenuous exercise. Supervised physical exercises, as part of a rehabilitation program, may be recommended^20^ after introduction of specific pharmacological therapy.

Studies in the 80s^21^ suggested a beneficial effect of anticoagulation based on the occurrence of *in situ* thrombosis reported in studies on lung biopsies in PAH patients. However, subsequent data^22^ have indicated a beneficial effect of anticoagulation only in patients with idiopathic PAH, heritable PAH or PAH associated with the use of anorectic drugs. Since then, indication for anticoagulation has been made based on a case-by-case risk and benefit analysis.^23^

#### Vasoreactivity testing

For patients with idiopathic, heritable, or drug-induced PAH, vasoreactivity test should be performed during diagnostic right heart catheterization. Inhaled nitric oxide (NO) at a dose of 10-20 ppm for 10 minutes is recognized as the gold-standard method to assess pulmonary vasoreactivity in patients with PAH. A positive vasoreactivity test is a fall of at least 10 mmHg in MPAP, fall to an absolute MPAP less than 40 mmHg, and unchanged cardiac output.^2^ Vasoreactivity testing aims to identify a subgroup of patients in whom the increase in vascular tone is the main mechanism of the genesis of PAH, rather than vascular remodeling.^24^

Patients with PAH that show a positive response to the vasoreactivity test should receive treatment with calcium channel blocker (CCB), preferably long-acting CCB at the highest tolerable dose.^25^ Administration of 10 mg amlodipine once a day, 30 mg nifedipine twice a day, and 60 mg diltiazem three times a day has been recommended. Patients who do not show improvements in functional class (to I/II) or in hemodynamic function should receive specific medications for PAH. Although nearly 12.6% of patients diagnosed with idiopathic PAH show an acute response to vasodilation, half of these patients do not show good clinical response to CCB at one year of follow-up.^26^ Patients who have not undergone vasoreactivity test should not be treated with CCB.^23^

## Specific treatment

### Treatment pathways

The main anatomical and physiological feature in PAH is pulmonary vascular remodeling, with development of intimal and medial thickening.^27^ Vasoconstriction also plays a role in the development of the disease, because of the increase of vascular tone and proliferation of smooth muscle cells (SMC) in arterioles.^25^ Three pathophysiological pathways related to molecular mechanisms involved in these histological findings have been identified and been used as pharmacological targets for the treatment of PAH – the prostacyclin (PGI_2_), pathway, the endothelin pathway, and the NO pathway.^28^

### The prostacyclin pathway

PGI_2_, a derivative of arachidonic acids, acts on transmembrane receptors of multiple tissues involved in many biological activities.^29^ In the pulmonary circulation, by stimulation of the IP receptor, PGI_2_induces relaxation of SMC in pulmonary arterioles leading to vasodilation, and inhibits their proliferation by stimulation of cyclic AMP synthesis.^30^ On the other hand, another molecule of the PGI_2_pathway, the thromboxane A2 (TXA2), counterbalances the PGI_2_effects, causing vasoconstriction and increase of platelet aggregation. In PAH, the levels of PGI_2_and the enzymatic activity of prostacyclin synthase are reduced, and the balance is shifted towards TXA2.^31^ Therefore, one of the strategies for the treatment of PAH is the direct action on IP receptor, by administration of prostacyclin (epoprostenol)^32^ or of a structural analog of TXA2 (e.g. treprostinil, beraprost and iloprost).^33 , 34^ Other drugs with different structures can also act on the IP receptor, like the selexipag.^35^

### The endothelin pathway

Endothelin-1 (ET-1) is the most potent natural vasoconstrictor of the biological systems.^36^ ET-1 levels are elevated in both pulmonary vascular endothelium and in the blood of patients with PAH.^37^ ET-1 exerts its physiological effect via two receptors, endothelin A receptor (ETA) and endothelin B receptor (ETB). Bosentan^38^ and macitentan^39^ are two endothelin receptor antagonists that non-selectively block ETA and ETB, and ambrisentan^40^ exhibits a higher affinity for ETA. These three drugs have been shown to be effective in the treatment of PAH.

### The nitric oxide pathway

NO is a potent endogenous vasodilator that acts in the SMC by stimulation of guanylyl cyclase (GC) and synthesis of cyclic GMP.^41^ Patients with PAH have reduced low levels of serum and tissue NO.^42^ The phosphodiesterase type 5 (PDE-5) is an enzyme responsible for degradation of cyclic GMP. Inhibition of PDE-5 leads to an increase in cyclic GMP levels and consequent relaxation of SMC and vasodilatation.^43^ Sildenafil, tadalafil and vardenafil are the PDE-5 inhibitors currently available.

Targeting another part of the NO pathway, riociguat, a GC stimulant, potentializes GC activity independently from NO.^44^ GC, stimulated by riociguat, potentializes the conversion of GTP into cyclic GMP and promotes vasodilation. Riociguat can be used for treatment of both PAH^45^ and chronic thromboembolic pulmonary hypertension.^46^ The drugs commercially available for the treatment of PAH in Brazil are listed in Table 2 .

**Table 2:** – Authorized drugs for the treatment of pulmonary arterial hypertension, available in Brazil

Drugs	Posology	Administration route	Common side effects	Pathway	Reference
Iloprost	2,5-5 mcg 6-9x day	Inhaled	Cough Local irritation	PGI_2_	^34^
Selexipag	200-1600 mcg 2x day	Oral	Headache Diarrhea	PGI_2_	^35^
Ambrisentan	5-10 mg 1 x day	Oral	Anemia Edema	ET1	^40^
Bosentan	62,5-125 mg 2x day	Oral	Anemia Hepatotoxicity	ET1	^38^
Macitentan	10 mg 1x day	Oral	Anemia Hepatotoxicity Edema	ET1	^39^
Sildenafil	20-80 mg 3x day	Oral	Headache	NO	^43^
Riociguat	0,5-2,5 mg 3x day	Oral	Headache Hypotension	NO	^45^

*PGI_2_: prostacyclin; ET1: endothelin-1; NO: nitric oxide.*

## Strategies of treatment

Over recent years, there has been a marked change in the use strategies of different drugs available, with trends towards earlier treatment and combination of different drug pathways/classes.

The combination of drugs is based on the potential synergy between these drugs acting simultaneously in different pathophysiological pathways. As compared with monotherapy, the addition of a second drug (sequential combined therapy) has shown to be beneficial for different combinations of drugs.^35 , 39 , 47^ These findings were corroborated by a meta-analysis of 14 studies on sequential combined therapy, showing a reduction in clinical worsening compared with monotherapy.^48^

In a different approach, a large clinical trial evaluated the use of ambrisentan plus tadalafil since the diagnosis of PAH.^47^ This drug combination caused a 50% reduction in the combined endpoint of clinical worsening when compared with the use of any of the other drugs alone, with no significant differences in side effects.

Although there are no studies directly comparing initial versus sequential combination therapies, evidence has suggested that initial combined therapy is well tolerated and beneficial even in patients classified as low risk.^47^ Thus, the current recommendation is to consider combined oral therapy since diagnosis as detailed in Figure 2 .

**Figure 2:** – *Treatment algorithm and risk stratification in the follow-up of pulmonary arterial hypertension proposed by a work group on pulmonary circulation (Heart Institute [INCOR], University of Sao Paulo). CCB: calcium-channel blockers; FC: functional class; 6MWD: six-minute walk distance; BNP: brain natriuretic peptide; MRI: magnetic resonance imaging; ECHO: echocardiography; CPT: cardiopulmonary test; ERA: endothelin receptor antagonists; PDE5i: phosphodiesterase type 5 (PDE-5).*

However, a minority of patients could still benefit from monotherapy strategy. Patients with portal hypertension, HIV, complex congenital heart disease, veno-occlusive disease, idiopathic PAH, and those with high likelihood of left heart failure and preserved ejection fraction can begin treatment with one drug class. Besides, long-term clinically stable patients in monotherapy may not need combination therapy.^23^

The potential of combination of three drugs since diagnosis has also been evaluated. A French retrospective study evaluated the efficacy of a triple combination therapy (epoprostenol, bosentan and sildenafil) in the initial treatment of severe PAH, and showed an improvement in NYHA functional class, exercise performance, and hemodynamic parameters. The study also showed longer survival of patients under triple therapy compared with that expected by the French historical registry, suggesting a long-term benefit of this approach.^49^

Nevertheless, for less severe patients, there is no evidence supporting the efficacy of initial triple therapy. A recent study evaluated the efficacy and safety of initial triple oral therapy with selexipag, macitentan and tadalafil versus initial double oral therapy with macitentan and tadalafil in 247 newly diagnosed PAH patients.50 No difference was found in terms of improvement of PVR, six-minute walk distance and N-terminal pro-brain natriuretic peptide (NT-proBNP) levels. Also, adverse effects were more common in the initial triple therapy group.

Triple combination therapy for the treatment of PAH patients seems efficient. The addition of selexipag as a third agent in patients receiving endothelin receptor antagonist and PDE-5 inhibitor was associated with less hospitalizations and events of disease progression.51 The beneficial effects were more pronounced in patients with World Health Organization functional class II.

Another potential strategy is the substitution of drugs targeting the same biochemical pathway. However, although promising, this strategy still lacks robust scientific evidence and should not be performed routinely.^52^

## Risk stratification and clinical follow-up

Assessing the risk of progression of PAH is essential to guide treatment. Strategies for mortality risk stratification evaluating the combination of multiple markers ( Table 3 ), both at diagnosis and during treatment, have shown effective in predicting the clinical course of the disease.^53 - 55^

**Table 3:** – Prognostic factors in pulmonary arterial hypertension (adapted from reference 8)

Prognostic determinants	One-year mortality (estimated)		

	Low risk < 5%	Intermediate risk 5-10%	High risk > 10%
Clinical signs of right ventricular failure	Absent	Absent	Present
Progression of symptoms	None	Slow	Fast
Syncope	None	Occasionally*	Repeatedly **
WHO FC	I, II	III	IV
6M-WD	> 440 m	165-440 m	< 165 m
Cardiopulmonary exercise test	Peak VO_2_> 15 mL/min/Kg (>65% predicted ) VE/VCO_2_slope < 36	Peak VO_2_11–15 mL/min/Kg (35-65% predicted) VE/VCO_2_slope 36–44.9	Peak VO_2_< 11mL/min/Kg (<35% predicted) VE/VCO_2_≥ 45
NT-proBNP plasma levels	BNP < 50 ng/l NT-proBNP < 300 ng/mL	BNP 50–300 ng/l NT-proBNP 300–1,400 ng/L	BNP > 300 ng/L NT-proBNP > 1,400 ng/L
Imaging tests (ECHO, chest magnetic resonance)	Right atrial area < 18 cm^2^ Absence of pericardial effusion	Right atrial area 18–26 cm^2^ Absent or minimal pericardial effusion	Right atrial area > 26 cm^2^ Presence of pericardial effusion
Hemodynamic parameters	Right atrial pressure < 8 mmHg CI ≥ 2.5 L/min/m^2^ ScvO_2_> 65%	Right atrial pressure 8–14 mmHg CI 2.0–2.4 L/min/m^2^ ScvO_2_60–65%	Right atrial pressure > 14 mmHg CI < 2.0 L/min/m^2^ ScvO2 < 60%

* Occasional syncope during brisk or heavy exercise, or occasional orthostatic syncope in an otherwise stable patient; ** Repeated episodes of syncope, even with little or regular physical activity. ECHO: echocardiogram; peakVO2: oxygen consumption at peak exercise; VE: ventilatory equivalents; VCO2: volume of exhaled CO_2_; BNP: brain natriuretic peptide; NT-proBNP: N-terminal pro-brain natriuretic peptide; CI: cardiac index, ScvO2: Central venous oxygen saturation; WHO: World Health Organization; 6M-WD: six-minute walk distance.

There are several methods to assess the risk of progression of PAH. One of them is to assign a score (from 1 to 3) to each variable, according to the range risk group (low, intermediate, or high, respectively). The overall risk is estimated by dividing the sum of all points by the number of variables. The nearest whole number classifies the risk as low (1), intermediate (2) or high (3) risk.^55^ A simpler approach aims to identify only patients at low risk, by using the combination of the following parameters: brain natriuretic peptide (BNP) levels < 50 pg/mL, six-minute walk distance > 440m and functional class of II.^53^ A third approach, derived from the REVEAL (Registry to Evaluate Early and Long-Term PAH Disease Management Registry) risk score calculator, includes up to 12 variables and has been recently updated.^56^

Although a more comprehensive approach can be superior to a more simplified evaluation, the low availability of the different tests limits their application. Thus, risk stratification, here suggested, is based on what is considered the minimum requirements for an adequate clinical management of patients with PAH, by a combination of functional class, six-minute walk test and BNP for identification of patients at “low risk” and “not low risk” ( Figure 2 ). According to their availability, tests like magnetic resonance, echocardiography and cardiopulmonary stress test should be encouraged due to their potential adjuvant role.^23 , 57^ The main objective of PAH treatment is to achieve or maintain a low risk of disease progression using the strategies and risk stratification above discussed.

## Particularities of different types of PAH

### PAH associated with connective tissue disease

PAH is a known complication of connective tissue disease (CTD). The main CTDs associated with PAH are systemic sclerosis (SS), systemic erythematosus lupus (SEL), and mixed CTD (MCTD), and in a lesser degree, dermatomyositis, and Sjögren syndrome.^58^ In Brazil, PAH associated with CTD represents nearly 25%^59^ of PAH cases. The primary disease associated with PAH is SS in western countries, and SEL in Asian,^60 - 62^ and the prevalence of PAH in SS and SEL patients is around 10%,^63^ and 4%, respectively.^64^

The presence of antiphospholipid, anti-RNP and anti-Ro antibodies is predictive of PAH in SEL patients.^65^ In patients with SS, the presence of long-term disease, telangiectasias, anti-centromere antibody positivity and reduction in the diffusing capacity for carbon monoxide (DLCO) are the main factors related to PAH.^66^

SS is characterized by an association between vasculopathy and tissue fibrosis.^67^ Clinically, SS is marked by a high prevalence of interstitial pulmonary disease (up to 50% of patients),^68^ and cardiac involvement (50-80% of patients), frequently leading to diastolic dysfunction.^69^ Therefore, in these patients, PH may be a result of either a pulmonary vascular disease alone or a combination of different pathophysiological mechanisms related to PH in groups II and III.^68^ Besides, these patients are at increased risk of pulmonary thromboembolism and associated veno-occlusive disease, which may even worsens patients’ prognosis.^68^ It is imperative to determine the preponderant mechanism to guide treatment of these patients.

In SS patients, annual screening for PH is recommended, even in asymptomatic patients.^70^ Screening models including risk factors, DLCO and BNP seem to be more sensitive than echocardiography alone.^71^ In SEL, due to the low prevalence of PH, the screening for this condition is not recommended as a routine practice, and echocardiography should be performed only for symptomatic patients ( Table 4 ).

**Table 4:** Risk factors and screening for pulmonary hypertension in patients with connective tissue disease

	Risk factors	Screening
SS	Long term disease Telangiectasias Anti-centromere Reduced DLCO or FVC/DLCO>1.6	-Annually in asymptomatic patients Echocardiogram ± biomarkers/ PFT or DETECT algorithm BNP/NTproBNP and PFT ± ECHO
LES	Serositis Antibodies: Antiphospholipid, RNP and Ro	In symptomatic patients only

*SS: systemic sclerosis; SEL: systemic erythematosus lupus; PFT: pulmonary function test; DLCO: diffusing capacity for carbon monoxide; forced vital capacity (FVC); BNP: brain natriuretic peptide; NT-proBNP: N-terminal pro-brain natriuretic peptide.*

**Table 5:** Treatment of pulmonary arterial hypertension associated with connective tissue

	Immunosuppression (IS)	Specific therapy
SS	Not recommended	According to risk stratification
SEL functional class I/II	CCP and GCT for six months	If PH continues after IS
SEL functional class III/IV	CCP and GCT plus specific therapy	Associated with IS, according to risk stratification
MCTD functional class I/II	CCP and GCT for six months	If PH continues after IS
MCTD functional class III/IV	CCP and GCT plus specific therapy	Associated with IS, according to risk stratification

*IS: immunosuppression; SS: systemic sclerosis; SEL: systemic erythematosus lupus; MCTD: mixed connective tissue disease; CCP: cyclophosphamide; GCT: glucocorticoid; PH: pulmonary hypertension.*

In case of clinical suspicion, the diagnostic flow is the same, aiming at differentiating the diagnosis of PAH (group I) from other forms of PH. Vasoreactivity testing is not applied for patients with DTC, as at least 1% of them have a sustained response.

For patients with inflammatory diseases, such as SEL and DMTC-PAH, immunosuppression is an alternative approach before specific therapy is initiated. In patients with functional class I and II, it is recommended to start treatment with cyclophosphamide (CCP) and glucocorticoid (GCT), whereas in those with functional class III and IV, a combination of CCP, GCT with specific therapy is recommended, due to a lower chance of response to immunosuppression, with reassessment after six months of CCP ( 5).^58 , 72^ In patients with SS, there is no evidence of improvement with immunosuppression.^73^

### PAH associated with HIV

The association between human immunodeficiency virus (HIV) and PAH was first described in 1987.^74^ Today, the estimated prevalence of PAH in HIV patients is 0.5%,^75^ and HIV infection is an important etiology of PAH in referral centers. In a Brazilian registry, 4.5% of PAH patients had HIV diagnosed.^59^

The pathophysiological mechanisms involved in the association between HIV and PAH are not fully understood. From a histological point of view, concentric laminar intimal fibrosis, medial hypertrophy, and plexiform lesions were found in up to 78% of patients, which resembles the changes observed in patients with PAH.^76^

The presence of PAH worsens the prognosis of HIV patients. CD4 counts lower than 200 cells/µL and a cardiac output lower than 2.8 L/min per m2 are independent predictors of survival.^77^ Then, antiretroviral therapy (ART) is recommended for all patients with HIV, regardless of CD4 count, viral load,^78^ or concomitant PAH.

With respect to the NO pathway, there are no controlled studies with sildenafil evaluating therapy response in patients with HIV and PAH. Positive results regarding exercise performance, functional class, and hemodynamic parameters were obtained from case studies.^79^ Caution is advised because of the interaction with ART, particularly with protease inhibitors.^80^ Also, there are no studies with riociguat, despite recent publication showing good safety and tolerability of riociguat and ART combination regimens.^81^

Among the endothelin receptor antagonists, bosentan was evaluated for the treatment of 59 HIV patients with PAH in a prospective study.^82^ There was an improvement in six-minute walk distance, symptoms and hemodynamic status. In pivotal studies on ambrisentan^40^ and macitentan,^39^ 17 and 10 HIV patients with PAH were included, respectively, and no interactions with ART were observed.^78^

Small case series evaluated prostanoids for treatment of HIV-PAH patients. Treatment with epoprostenol resulted in improvement of hemodynamics,^83^ and iloprost improved functional lass and six-minute walk test in four patients.^84^ In a pivotal trial, selexipag was administered to 10 HIV patients.^35^ In a study evaluating potential interactions between ART and selexipag, no interaction was detected, and adaptations of the selexipag dose were not required in a study with healthy subjects treated with a single dose of 400 µg selexipag in combination with multiple doses of lopinavir/ritonavir (400/100 mg) twice daily.^85^

### Portopulmonary hypertension

Portopulmonary hypertension (PPH) refers to a form of PAH that is associated with portal hypertension. Portal hypertension leads to the formation of portosystemic shunts and increased pulmonary blood flow, which results in endothelial remodeling and dysfunction, with elevations in ET-1 production and pulmonary vascular pressure.^86^

It is estimated that 10.6% of PAH patients have PPH,^87^ and in a review^88^ of 1,205 consecutive liver transplant patients, the incidence of pulmonary hypertension 8.5% of liver transplant candidates. PPH is not associated with the etiology of portal hypertension nor with the severity of lung disease.^89^

Clinical presentation of PPH is similar to other forms of PAH, and the most common symptom in PPH patients is dyspnea. In PAH, the presence of ascites may be indicative of PPH.^90^ For diagnosis of this condition, right catheterization can be made to confirm PAH, as described before, and portal hypertension can be confirmed by a hepatic venous pressure gradient greater than 4 mmHg.^91^

The treatment of PPH was evaluated by small studies, that demonstrated beneficial effects of three classes of drugs. Hepatotoxicity of endothelin receptor antagonists may not be a major problem in continuously monitored patients.^92^ The largest study conducted with PPH patients showed hemodynamic improvements after 12 weeks of macitentan, with no hepatic side effects.^93^ Some well-established drugs for the treatment of portal hypertension, including beta-blockers, should not be administered in PPH, due to worsening of hemodynamic status and exercise capacity.^94^

It is worth pointing out the role of PPH in liver transplant candidates. PPH is a marker of a worse prognosis in the perioperative period, and MPAP values lower than 35 mmHg indicated a lower risk of death in liver transplant recipients.^91^ Therefore, a screening echocardiogram to detect PAH is mandatory for patients in the liver transplant waiting list and for patients with portal hypertension presenting with (even mild) dyspnea.^95^

### Schistosomiasis

Schistosomiasis (Sch) is an endemic parasitic disease in Brazil, associated with poverty and poor sanitation. It is caused by trematode worms of the genus Schistosoma, and its transmission has been documented in more than 70 countries. Although the hepatosplenic form is the most common manifestation of chronic Sch,^96^ one of the most severe and limiting manifestations of the disease are related to impairment of the pulmonary circulation in the presence of PAH (Sch-PAH).

Nearly 5% of patients with the hepatosplenic form of Sch have PAH.^96^ Thus, considering the high prevalence of schistosomiasis worldwide, Sch-PAH is potentially one of the most prevalent PAH, especially in developing countries.^97^ A recent registry showed that Sch-PAH can represent about 20% of the incident cases of PAH.^59^

The diagnosis of Sch-PAH requires invasive confirmation of PAH (see “definition” session) and confirmation of hepatosplenic Sch, with presence of ultrasound changes compatible with the disease (e.g., periportal fibrosis) plus one of the following epidemiological factors: 1) patients from endemic areas; OR 2) previous treatment for Sch; OR 3) detection of Schistosoma eggs in stool samples or rectal biopsies.^98^

Although Sch-PAH usually have a better clinical course than idiopathic PAH, even in the absence of specific therapy, it does not mean that the disease poses no risk. Sch-PAH mortality can reach 15% in three years.^99^ Cases of PAH with pronounced dilatation of pulmonary arteries should suggest Sch-PAH, especially in areas where Sch is highly prevalent.^100^

Treatment of Sch-PAH is similar to the other forms of PAH. Cases series have demonstrated clinical and hemodynamic benefits,^101^ in addition to longer survival with specific therapy.^97^ Thus, also for Sch-PAH, it is recommended to follow the therapeutic algorithm as proposed in Figure 2 . Anticoagulation should be avoided due to the risk of upper gastrointestinal bleeding. All patients should receive at least one cycle of anti-parasite treatment, since it is not known whether the persistence of infection can contribute to progression of pulmonary vascular dysfunction.^97^

### Congenital heart disease

PAH is a relatively common condition in patients with congenital heart diseases (CHDs), affecting 5-10% of this group.^102^ CHDs can cause pulmonary hyperflow, inducing endothelial remodeling and consequent PVR, which is histologically indistinguishable from other forms of PAH. Thus, most cases of CHD-PAH are classified as group I. In this group, when PVR exceeds the systemic vascular resistance, the direction of the shunt reverses, which characterizes the Eisenmenger syndrome.^103^ Patients with this syndrome have severe hypoxemia, and hematological changes (erythrocytosis and low platelet count). Also, these patients may have hemoptysis, stroke, brain abscess in addition to a higher incidence of sudden death.^9^

The treatment of CHD-PAH should follow the guidelines previously established for other forms of group I. Therapeutic phlebotomy can be considered for Eisenmenger syndrome patients who present hyperviscosity symptoms (usually hematocrit levels above 65%).^9^ As in other forms of PAH, anticoagulation is controversial in Eisenmenger syndrome,^104^ and should be weighed case by case. Likewise, the benefit of supplemental oxygen in patients with Eisenmenger syndrome and hypoxemia is questionable,^105^ and indication should be tailored to the patient’s needs.

With respect to the specific therapy for CHD-PAH, the only validated drug treatment was bosentan, which improved the six-minute walk test and decreased PVR after 16 weeks of treatment in functional class III patients.^106^ Smaller series have shown beneficial effects with PDE-5 inhibitors and prostanoids.^9^

Some CHDs, including patent ductus arteriosus, sinus venosus atrial septal defect, or anomalous pulmonary venous drainage can be unnoticed in PAH patients, who would be considered as idiopathic PAH. However, in the context of PAH, the diagnosis of a CHD in the context may indicate the need for surgical repair of the defect, according to PCR at diagnosis^107^ and the clinical course. This evaluation should be performed in centers with expertise in this condition for a proper referral (or not) for surgery. The mere possibility of correction reinforces the necessity of actively investigating the presence of CHD. In patients undergoing diagnostic evaluation, this can include echocardiography with microbubbles for examination of intracardiac and extracardiac shunts.

In addition, some complex, rarer CHDs, are classified as group V, i.e., of uncertain or multifactorial mechanisms.^8^ In these cases, due to the absence of prospective studies, the pharmacological or surgical approach (including heart-lung transplantation) should be individually performed.

## Special situations

### Pulmonary hypertension and pregnancy

Physiological changes in pregnancy, including increase in blood volume and cardiac output,^108 , 109^ are generally not well tolerated in PAH patients. Thus, pregnancy is associated with high morbidity and mortality rates, varying from 30 to 56%.^18^

Thus, because of the high lethality of PAH in pregnancy, it is recommended the use of two contraceptive methods, although there is no consensus on the best method. Barrier methods are considered safe, although their efficacy is directly related to a correct use.^108^ Hormonal methods of birth control, as those containing progestin only, are effective; combination with estrogen should be avoided due to the increased risk in thromboembolic events.^109^ Intrauterine devices may be used, but vasovagal reactions may occur at their insertion.^110^

When pregnancy is confirmed, the patient should be informed about potential risks. In extreme cases, therapeutic interruption of gestation may be considered,^109^ preferably before 22 weeks of gestational age.^111^ In a recent European registry of PH and pregnancy,^112^ therapeutic abortion was performed in 4% of cases, whereas perinatal mortality in idiopathic PAH was performed in 43% of the cases. Although there is no consensus about the safest birthing method, cesarean section at 32-36 weeks’ gestation, without general anesthesia, has been the preferable method.^111 , 113^ Preeclampsia or eclampsia, preterm birth, fetal death, postpartum bleeding were the most common complications in pregnant women with PH.^114^

Specific therapy should be adapted to pregnant women with PAH who do not accept pregnancy termination and insist on continuing with their pregnancy. Case series have demonstrated the efficacy of sildenafil and prostacyclin analogues in pregnancy.^18 , 113 , 115^ However, endothelin receptor antagonists should be avoided due to their potential teratogenic effect.^116^

### Angina and PAH

Angina is reported by 15.8^117^ -29.0%%^9^ of patients with PAH and may result by an imbalance between myocardial oxygen supply and demand in the right ventricle, with no change in the flow of epicardial coronary arteries. The increase in ventricular wall tension leads to a decrease in the coronary flow reserve, which, associated with greater ventricular work and oxygen consumption, and myocardial hypertrophy, results in ischemia. However, another consequence of PAH is vascular remodeling, with increased pulmonary artery diameter.

Dilated pulmonary artery may induce inferior displacement of the left main coronary artery (LMCA), which become in close contact with the left aortic sinus ( Figure 3 ). This compression may cause LMCA stenosis.^118^ A prevalence of up to 40% of LMCA occlusion caused by external compression has been described in PAH patients with angina.^117^ The main predictor of LMCA compression was a pulmonary artery diameter > 40 mm, followed by a pulmonary artery to ascending aorta diameter ratio ≥1.5.^117^

**Figure 3:** – *A) Computed tomography angiography showing inferior displacement of the left main coronary artery caused by an aneurysm of the pulmonary artery and left coronary ostial stenosis. B) coronary cineangiography of the same patient, showing occlusion of the left main coronary artery ostium.*

The prognostic impact of LMCA compression is still uncertain. Therapeutic management of this condition if similar to that performed in LMCA lesion caused by atherosclerosis: myocardial revascularization, with the percutaneous approach being the safest and most attractive modality for these patients.^119^ Data from an Italian study demonstrated good results with this approach. In a mean follow-up of 4.5 years of 53 patients who underwent angioplasty, 19 patients (37.3%) died, with no case of infarction or stent thrombosis, and five patients required a new angioplasty.^120^

LMCA compression should be considered in all patients with PH and angina, with initial investigation by computed tomography angiography. Patients with a high clinical probability of extrinsic compression of the LMCA (e.g., patients with large pulmonary artery aneurysm and angina), referred for right catheterization, should be submitted to coronary cineangiography, even without coronary computed tomography angiography. With the increase in survival of PAH patients, atherosclerotic comorbidity should also be considered, with control of traditional cardiovascular risk factors.

### Supraventricular arrhythmias and PAH

Cardiac arrythmias are frequent in PAH patients, notably supraventricular tachycardias, caused by either by increased automation or re-entry. Retrospective studies have reported an annual incidence of sustained supraventricular tachycardia between 2.8%^121^ and 3.5%.^122^ However, these numbers underestimate the real incidence, due to the lack of prospective studies with a strategic search and strategy of continuous monitoring or of accessible demand. These arrythmias are caused by right ventricular pressure overload, cardiac remodeling, and increased adrenergic tone, associated with PH. Also, hypokalemia and hypomagnesemia caused by chronic treatment with diuretic may also be a cause of arrythmias.

The most common sustained arrythmias are atrial fibrillation and atrial flutter, followed by atrioventricular-nodal reentrant tachycardia and sustained atrial tachycardia. Most patients have worsening of functional class, and persistence of arrythmia leads to higher mortality.^121^ The treatment of arrhythmias is focused on restoring cardiac rhythm by using drugs of modest inotropic effect (e.g., amiodarone), programmed electrical cardioversion, and in some cases, electrophysiological study and ablation (which should be early considered in atrial flutter). In preparation for cardioversion, or in cases of recurrence or cardioversion failure, monitoring of ventricular response is essential to guarantee cardiac output, and tailor the therapy according to the right ventricular functional reserve. Patients with important right ventricular dysfunction caused by pulmonary hypertension have poor tolerance to betablockers, and even the use of calcium channel blockers may result in clinical decompensation. In these situations, digoxin and amiodarone may be convenient. It is worth pointing out that all patients with PH and atrial fibrillation should receive anticoagulant therapy, due to the high risk of systemic and cerebral thromboembolism.

## Conclusion

In the last decades, important advances have been made in the treatment of PAH, a severe, progressive, incurable, and potentially fatal disease. For an adequate therapy, correct hemodynamic diagnosis and etiology classification are fundamental. Many etiologies – rheumatic disease, portal hypertension, CHDs, Sch – require specific measures, in addition to drug therapy for PAH. The specific therapy for PAH is based on medications that act on three pathophysiological pathways – prostacyclin, endothelin, and NO pathways. Today, it is recommended that initial treatment begins with a combination of two oral therapies and increases if patient does not achieve the desired therapeutic target, which was established based on the stratification of cardiovascular death risk.

Cardiovascular complications of PAH (LCMA compression, supraventricular arrhythmias) are common and should be promptly identified and treated, as they affect the quality of life and probably the prognosis of patients with PAH.
